# The Need to Appear Healthy: Concealment of Chronic Illness, Privacy, and Self-Sufficiency Among Chronically Ill Older Nigerians

**DOI:** 10.1093/geroni/igad141

**Published:** 2023-12-29

**Authors:** Kafayat Mahmoud, Tamara A Baker, Darlingtina Esiaka, Saliu Balogun

**Affiliations:** Center for Innovation in Social Science, Boston University, Boston, Massachusetts, USA; Department of Psychiatry, School of Medicine, University of North Carolina at Chapel Hill, Chapel Hill, North Carolina, USA; Center for Health Equity Transformation, University of Kentucky College of Medicine, Lexington, Kentucky, USA; Menzies Institute for Medical Research, University of Tasmania, Hobart, Tasmania, Australia

**Keywords:** Ageism, Health, Social networks, Well-being

## Abstract

**Background and Objectives:**

Prior research has highlighted the beneficial impact of social networks and social support on older adults’ physical and psychosocial well-being. However, the impact of the relationship between chronic illness and social networks on the psychosocial well-being of older Nigerians remains understudied. This study explored how older Nigerians with chronic illnesses navigate the physical, mental, and emotional changes due to their chronic disease diagnosis within their social contexts.

**Research Design and Methods:**

The current qualitative study used semistructured in-depth interviews with 19 purposively sampled older adults, aged 50 years and over, chronically ill, and receiving clinical care to examine the role of social networks in how chronically ill older Nigerians cope with their diagnosis.

**Results:**

Three main themes reflecting participants’ experiences emerged from the findings: (1) closely knit circles, (2) privacy and self-sufficiency, and (3) body image. Results show that chronically ill older Nigerians prefer to keep the knowledge of their conditions strictly within their close family circles. It was considered horrific to inform friends, community members, and religious groups about one’s chronic illness. Findings further reveal that the need to appear healthy to one’s social network stems from the fear of being discriminated against and attempts to maintain some level of normalcy when interacting with others. Additionally, feelings of inferiority and shame limited their participation in social activities and social network maintenance.

**Discussion and Implications:**

We discuss the implications of the results for the mental well-being and quality of life of chronically ill older Nigerians and make recommendations for policies and resources that can improve the well-being of chronically ill Nigerians.

**Translational Significance:** This study fills an important gap in the literature regarding social networks’ positive and sometimes unintended negative consequences on self-perceptions and the coping strategies chronically ill older adults adopt. Older adults in Nigeria face age-related and disease-related stigma that persists and limits their full participation in society. Navigating these realities could be very detrimental to their well-being. This study also highlights the need for policies and resources that can improve the quality of life of older adults as they navigate the sociocultural complexities associated with living with chronic conditions.

In Nigeria, an estimated 25 million people live with chronic illness(es), and studies have begun demonstrating the rising burden of diseases. For example, there are emergent data on increased risk and prevalence of diabetes (Kyari et al., 2014), hypertension, cardiovascular diseases (Adeloye et al., 2015), obesity (Aliyu et al., 2015), cancer (Chidebe et al., 2018), and chronic kidney diseases (Okoye et al., 2011), with increased susceptibility at older ages. Despite this evidence, limited attention has been directed toward understanding how older adults in Nigeria cope with chronic diseases and how the presence of the illness affects their psychosocial well-being. More specifically, little is known about how the experience of chronic disease affects social and community interactions of older adults in Nigeria.

A significant body of research has emphasized the salience of social networks for improving older adult health and well-being through the provision of social support, social control of health behaviors, companionship, and improvement of self-efficacy (House et al., 1988; Litwin, 1998). However, other research suggests that the onset of chronic illness negatively affects the continued maintenance of social network ties, especially with nonkin relationships (Qu, 2023). Indeed, prior research framing the sociocultural experience of chronic illness, for instance, in the United States and Europe, has highlighted the consequential impacts on changes in social relationships, specifically with regard to expectations in the exchange of social support and withdrawal from social relationships, as well as changes in self-perceptions (Bury, 1982; Kelly & Field, 1996).

Previous studies examining the social experiences of people diagnosed with diseases in Nigeria have largely focused on infectious diseases such as HIV/AIDS (Monjok et al., 2009; Weiss, 2008), and discussed how persons with communicable diseases deal with negative stigma and discrimination. The experience of stigma affects their ability to maintain social relationships and participate in social/communal activities (Monjok et al., 2009; Weiss, 2008). These social experiences of stigma and discrimination are principally influenced by cultural beliefs about the etiology and consequences of these infectious diseases, as well as the treatment-seeking behaviors. These cultural beliefs consequently inform people’s reactions when diagnosed with diseases. For instance, persons living with HIV/AIDS would hide their treatment cards from close family and community members because of the perception that they are spiritually inflicted with the disease and the fear of being exposed to discriminatory acts (Olalekan et al., 2014; Weiss, 2008).

However, limited studies have assessed older persons’ social experiences of chronic medical conditions within Nigeria’s sociocultural context. The current study aims to explore how chronically ill older adults in Nigeria perceive their chronic medical diagnosis(es) and their lived experiences within their familial and social contexts. The following research questions are central to this study: What is the association between social networks, chronic conditions, and older adults’ psychosocial well-being? And how do social networks influence perceived stigma among older persons with chronic conditions? Findings from this study may contribute to policy and guidelines to improve chronic disease management among older people in Nigeria.

## Method

### Study Design

A qualitative design with a constructionist-interpretivist approach was used to explore the participants’ experiences of chronic disease(s) and symptom-related outcomes. This constructionist-interpretivist approach allows us to explore participants’ experiences from their own perspectives (Ormston et al., 2013). Although the constructionist approach facilitated our understanding of how older people perceived their illnesses, the challenges they faced, and how they coped with their chronic conditions, the interpretive approach facilitated the understanding of older peoples’ explanations of their social realities (Ormston et al., 2013). Previous studies have employed this method to assess the influence of social support and religion in the maintenance of positive well-being among chronically ill older persons (e.g., Harvey & Silverman, 2007; Loeb, 2006). The Ethics Review Committee of a University in the United Kingdom and Kwara State Ministry of Health approved this study.

### Participants and Procedure

The study was conducted in Ilorin, the capital of Kwara State, in the North-Central geopolitical zone of Nigeria. Ilorin comprises three Local Government Areas (LGAs): Ilorin-East, Ilorin-South, and Ilorin-North LGAs. The people are predominantly of the Yoruba tribe and mostly traders by occupation. Participants were recruited from two healthcare facilities in Ilorin. Both facilities provide general specialty care for various diseases, including diabetes, glaucoma, hypertension, arthritis, stroke, and Alzheimer’s disease. Health facilities proved to be the most effective and accessible way of recruiting participants as opposed to social gatherings or marketplaces because it ensured that participants in the study had valid, medical doctor verified chronic disease status as opposed to subjective self-diagnosis, which would have compromised the purpose of the study. Purposive and convenience sampling were used to recruit participants for the study. This technique proved to be the most efficient recruitment strategy, particularly for this subpopulation of interest. Eligible participants were adults 50+ years of age, able to provide full consent, reported one or more chronic illnesses, and receiving treatment from one of the two study sites. Those who could not speak or give their full informed consent were excluded from the study.

Thirty older adults with a range of chronic conditions were invited to participate, however, only 19 agreed to participate in the study. Consent was obtained from those who met the eligibility criteria, and all interviews were conducted in designated and private areas of the hospitals. All interviews were conducted in Yoruba or English language, depending on the preference of the participant.

### Interview Protocol

A semistructured interview guide (see Supplementary Material) was designed to ensure that the interview questions directly addressed the research questions (Castillo-Montoya, 2016). Also, enough leeway was given to respondents to expand on their experiences and convey their interpretations of their experiences. Chronic medical diagnoses were assessed using a list of commonly occurring chronic illnesses. Respondents self-reported if a doctor diagnosed them with any of the following chronic illnesses: osteoporosis, renal or kidney disease, stroke, hypertension, heart attack or myocardial infarction, congestive heart failure, and arthritis. Demographic information, including age, sex, marital status, level of education, ethnicity, and religion, was also collected. All interviews were audio-recorded to ensure that responses were appropriately and efficiently attained.

### Data Analysis

Audio-recorded interviews were transcribed and saved on a password-protected computer. Data were manually coded and analyzed using thematic analysis. This was done to capture the most important information that participants continually emphasized during their interviews. Following the steps proffered by Braun and Clarke (2006), thematic analysis was conducted by familiarizing oneself with data, producing initial codes from the transcripts, searching for initial themes, reexamining themes, defining and naming themes, and producing the research report.

## Results

Nineteen respondents (5 men and 14 women), aged between 50 and 80 years, completed the study. Table 1 shows the demographic and social characteristics of the respondents. Respondents self-reported dealing with various diseases and, sometimes, comorbidities, including diabetes, hypertension, stroke, arthritis, and glaucoma. Participants were predominantly from the Yoruba ethnic group in Nigeria, and residents of Ilorin, Kwara State. They were primarily of low socioeconomic status with eight participants having no education, six had less than high school education, two participants had up to high school education, and two had tertiary education. Participants were also primarily traders (13 older adults), one plumber, two civil servants, two retired traders, and one older adult reporting no current occupation. Finally, with regards to living arrangements, all participants lived with their family members. Three key themes emerged and illuminated the participants’ experiences as they navigated interpersonal relationships within their social networks. The themes are closely knit circles, privacy and self-sufficiency, and body image.

**Table 1.** Demographic and Social Characteristics of Respondents (*N* = 19)

Participant number	Age	Sex	Marital status	Highest educational qualification	Nature of illness	Occupation
P1	67	Male	Married	Primary education	Hypertension	Retired
P2	58	Male	Married	No education	Hypertension	Trader
P3	80	Female	Widow	No education	Hypertension	Retired
P4	57	Male	Married	Primary education	Diabetes Hypertension	Plumber
P5	56	Female	Widow	Primary education	Diabetes Hypertension	Trader
P6	52	Female	Married	Primary education	Diabetes Hypertension	Trader
P7	55	Female	Widow	Primary education	Hypertension	Trader
P8	60	Female	Separated	No education	Diabetes	Fish seller
P9	53	Female	Married	Secondary education	Arthritis Hypertension	Trader
P10	50	Female	Widow	No education	Diabetes	Trader
P11	73	Female	Married	No education	Hypertension	Trader
P12	60	Female	Widow	No education	Diabetes Hypertension	Trader
P13	Don’t know	Female	Widow	Secondary education	Hypertension	Retired
P14	Don’t know	Female	Married	No education	Diabetes Hypertension	Trader
P15	Don’t know	Male	Widower	No education	Glaucoma	Unemployed
P16	62	Female	Widow	Primary education	Glaucoma	Trader
P17	51	Female	Widow	Tertiary education	Glaucoma	Civil Servant
P18	55	Male	Married	Primary education	Glaucoma	Businessman
P19	50	Female	Married	Nursing School	Glaucoma	Nurse

### Closely Knit Circles

Chronically ill older adults primarily rely on their families for emotional and instrumental support, and health information shared within family circles is kept strictly confidential. Most participants expressed being solely dependent on their extended family networks for sustenance and care. Also, their immediate family members were their sole confidants. For instance, when asked whether his family members knew of his condition and how they provided support, P1, a 67-year-old retired male trader with hypertension, responded: “Everybody knows. What kind of life is that if I am sick and my children do not know? That is not normal, and yes, my wife and children have supported me financially.”

However, this knowledge was mostly restricted to immediate family members. It was preferable that other people in their communities viewed them as healthy, without illness. It was important for them to portray a healthy and thriving life. This pretense to the “outsiders” ensured that they were not discriminated against or looked down upon and could enjoy equal statuses and privileges as other healthy members of the society. For the widows in the study, however, support was mainly from children perceived to have taken on the role of their “husbands” and, thus, provided financial and physical support to the older adults. For example, when asked how she coped with the expenses of her chronic illness, P16, a 62-year-old woman with glaucoma responded:

My children are my “husbands.” They support me, although one promised to send me the money for my drugs but has not. That is why I have been unable to buy my medications. I pray God enriches my children, so they can take care of me.

Similarly, P7, an older female participant aged 55, widowed, and diagnosed with hypertension declared:

When I explain to my children that I am going to the hospital, they know I would need drugs. I discuss with them, and they give me what they can, and I add the rest to it since I am also a trader. God should not make us unhappy; my children are my joy.

### Privacy and Self-Sufficiency

A closely related theme to close-knit circles, which emanated from the data is the importance of privacy and the appearance of being self-sufficient by respondents to their friends and community members. This theme differs from closely knit circles because it refers to how older adults guarded their health and other personal information and preferred to be seen as financially and physically capable and self-sufficient in meeting their own needs beyond receiving support from family. Respondents generally expressed that they were well taken care of and did not need interference from community members. For instance, when asked whether people in their communities and religious groups were aware of their conditions and had shown support in any way, P3, an 80-year-old widow suffering from hypertension and dependent on her sister-in-law for her up-keep declared:

There is no need because they know I am well taken care of. No one knows the nature of my illness, so they cannot render support. I attend church regularly; only sick people who are bedridden require support, you know, there is no problem at all.

Likewise, P5, another widow aged 56, and suffering from diabetes and hypertension said:

Ah, why? I am not bedridden. Except for people I have told, there is no need for that. Everyone has some little disease they are nursing, so … they have not come to greet me because I am not bedridden.

P7, a 55-year-old widow also stated:

Haha, when I am not bedridden. Once you attend immediately to whatever is ailing you, they cannot know. So, you know … my group members, there is not many of us, whenever we meet, we keep to ourselves, discuss whatever we need to and the disperse. Also, since it [the disease] is not so serious, I did not inform anybody to the extent that they will need to visit me at home.

Apart from wanting to maintain some level of privacy and self-sufficiency, other reasons provided for limited support from friendship and community networks were the economic hardships being experienced by everyone in Nigeria. Participants generally opined that everyone had their own problems and, as such, could not be burdened by other people’s problems. Respondents also needed to pretend they were not sick when sick to save face amongst their community members and continue with regular daily activities without interruption. So, until there are physical signs that are difficult to deny, individuals are most likely to keep their chronic disease conditions secret and carry on with regular daily activities. Furthermore, these statements suggest a clear distinction between the home and public spheres, with respondents distinguishing between those they could confide in and those they could not. Thus, implying that chronically ill older persons value their privacy and self-esteem.

Conversely, some received support, although limited, from their friends, religious groups, and community members. These are individuals who had been placed on admission in the hospitals due to their critical states. For instance, P2, a 55-year-old male trader diagnosed with hypertension said:

They come to visit me and contribute money to give to me. I also go to “asalatu” [prayer group]. When I went [to the prayer group] last Saturday, they expressed concern that I looked a little lean and I explained to them that it was because my prescriptions had changed.

### Body Image

An important recurring theme in the interviews was how chronically ill respondents viewed themselves, their concerns regarding their physical appearance, and how they managed their public image. It was clear that they wanted to be completely healthy and not dependent on medications. It was also evident from the interviews that respondents felt they looked worse than others and thus felt inferior or even ashamed, which prevented them from participating in social activities as much as they would have wanted. Additionally, participants were very sensitive to other’s comments and reactions to their changing physical appearance, such as looking leaner or older. And, although some of the comments were from people who did not have prior knowledge of respondents’ chronic condition, it was enough reason for them to limit their participation in social activities, or even appear publicly. For instance, P4, a 57-year-old plumber dealing with diabetes and hypertension, when explaining whether he was still very active socially, stated:

Next week Saturday, there is a carnival in my village. I even mentioned that I would like to go but my wife responded, “Do you want to go there looking like this? You have not recovered to the point that you can travel.” That’s why I decided to rest at home.

When asked to clarify what his wife meant by how he looked, he explained:

She meant I had lost too much weight to travel right now and that it was better I get some rest and not think about travelling. Look at my photograph taken in April [shows an old photograph], who would have known that I had lost weight, compare that with how I look right now. I went to work yesterday, and people commented that I’m older now, but I told them it’s not old age. I know what’s wrong with me, and it’s this disease [diabetes], but because they saw that I had lost weight, they assumed it was because of old age.

Similarly, a P14, a female respondent with diabetes and hypertension explained:

I just kept losing weight and everyone kept asking me why I was losing weight. Sometimes when I see people walking on the streets, I say to myself “O God, it is drugs that are keeping me alive, without them I won’t survive. I cry but I don’t let my children know because once they see me crying, they will also cry.

The respondents reported common tendencies to affirm the existence of their illness by other people—who commented about their physical appearances and made insinuations about their weight loss or gain, not looking healthy or looking older. These comments negatively affected their self-esteem, limiting their social activities for fear of inviting similar comments about their physical appearances from other people. This theme explains that families, friendship networks, and community membership could also have negative influence on an older persons’ emotional well-being.

## Discussion

This study explored sociocultural experiences that influence coping strategies adopted by chronically ill older adults in Nigeria. The study investigated how social networks, such as the family, community members, and religious groups, influenced the coping strategies adopted by chronically ill older adults. Given the growing population of older persons, the rising incidence of chronic conditions, and the inadequate health infrastructure for managing chronic illnesses among the Nigerian populace, we must understand how older people utilize their personal and social resources to cope with chronic illness diagnoses. Such understanding will help identify appropriate resources and policies to improve the well-being of older persons as they navigate the complexities of living with chronic illness. This study fills an important gap in the literature regarding the positive and sometimes unintended negative consequences of social networks on coping strategies adopted by older persons dealing with chronic illness in North-Central Nigeria. The study findings were derived from semistructured interviews of 19 purposively selected older adults dealing with chronic conditions in North-Central Nigeria. Participants in this study were also predominantly of low socioeconomic status, most with little to no education, which may have influenced their experiences. Below, we discuss the findings of this study in relation to previous studies and with consideration for the context within which the study was conducted.

We found that the family, as a social support network, was the most important unit in helping older persons cope with chronic illness diagnoses. Respondents expressed that they depended on their families for their upkeep and emotional well-being. Because chronically ill older adults often live with a loss of functional ability, independence, autonomy, and mental ability, reliance on family for physical and emotional support can be regarded as a critical part of care. As such, families become a valuable coping resource. Additionally, we found that for widowed, chronically ill older adults, adult children were central to their pathways to utilizing healthcare facilities and services. The adult children were instrumental in initiating and ensuring continued access to healthcare and financial resources for managing chronic conditions. This finding supports earlier studies (e.g., Litwin & Stoeckel, 2014; Mahmoud et al., 2022; Park et al., 2015). Congruent with our findings, these studies underscored the importance of families for the healthy coping processes utilized by chronically ill older adults.

However, our study also revealed that family members are not always able to support their chronically ill older persons due to financial hardships. This helps to illuminate the salience of financial stability in promoting familial support for chronically ill older adults. Interestingly, our findings revealed that chronically ill older persons might also be breadwinners in their families. Even while dealing with their chronic conditions, they are still responsible for the upkeep of others in the family. Thus, they are often thrust into an unending burden of caring for their families and their chronic illnesses while struggling to strike a balance between the two. This was a novel finding in our study that was not evident in any of the earlier reported studies, which investigated the role of families in the coping process for older adults with chronic disease (e.g., Litwin & Stoeckel, 2014; Park et al., 2015).

The study findings also showed that religious groups sometimes augment family support by providing instrumental support, emotional care, and financial assistance to chronically ill older persons. For example, religious group members visit and pray for the sick to alleviate their worries. In addition to the role of religious groups in providing support, being religious appears to be instrumental in maintaining well-being and coping with chronic illness among older adults. Similar to previous studies (e.g., Mahmoud et al., 2023), the study findings indicate that religion equips chronically ill older adults with a psychological outlook for their illness and helps them cope positively. Their responses show that the respondents employ religious coping to handle the uncertainty and difficulties associated with their diagnoses. By keeping a positive outlook, the participants could maintain a level of well-being that buffers negative psychological experiences associated with their diagnosis.

Further, this study revealed that chronically ill older adults preferred to keep knowledge of their illness strictly within their close family circles, except in extremely dire circumstances. It is almost a taboo to inform community members, friends, and religious groups about one’s chronic illness. Some reasons for the need to appear healthy to others might stem from shame of having a chronic illness and the fear of being pitied or stigmatized. This finding underscores prior studies highlighting tendencies for people to hide their illness from others to protect themselves from social repercussions and maintain their well-being (Weiss, 2008). By keeping information about their chronic illness within close family units, the respondents were able to control the narrative about their lived experiences and interact with other community members as if they were undiagnosed with illness. This tendency to maintain some level of normalcy and continued participation in social activities helped chronically ill older adults maintain desirable levels of social status and presence within their communities. In turn, the strategy of secrecy appeared to improve the mental well-being of chronically ill older adults, especially those wary of how they will be perceived if the news of their illness gets out in their community.

Although Litwin (2007) found that friendship networks and community participation were essential to older persons’ well-being. For the older adults in this study, concealing their chronic illness diagnoses is important for their well-being and continued community participation. Additionally, the finding suggests that families, friendship networks, and community members could also negatively influence the emotional well-being of chronically ill older adults. For instance, respondents commented on how family members, friends, and community members made negative statements about their physical appearances. These statements resulted in respondents having low self-esteem and feeling anxious about how they look when around others and thus, consequently, affected their participation in social activities.

Our study adds to earlier findings that showed older adults with chronic illness tend to hide their illness from others. On the one hand, they are faced with an age-related stigma that persists and serves to limit their full participation in society. On the other hand, they face disease-related stigma that is very common in Nigerian communities (Adebiyi et al., 2015). Although stigmatization and discrimination against persons with communicable diseases have been documented extensively in research (e.g., Monjok et al., 2009; Olalekan et al., 2014), it is surprising that people dealing with noncommunicable chronic diseases share somewhat similar perceptions of themselves.

### Policy Implications and Recommendations

This study examined the association between social networks, chronic illness, and psychosocial well-being among older adults. The health and well-being of older persons should be prioritized, and chronic illness, projected to continue to increase and affect an appreciable proportion of the older population, should also be a major focus. Provisions should be made to ensure that older persons dealing with chronic illnesses receive free and accessible care available in healthcare centers in the country.

There should also be nationwide campaigns by the government and nongovernmental organizations to instigate community education and mobilization. Community education would be directed toward providing information about different chronic diseases, risk factors, signs, and symptoms and encouraging positive healthcare behaviors among members of society. Increased awareness of chronic diseases would result in increased inclusiveness where everyone is aware of what chronic disease(s) their community members are suffering from, reduced stigmatization, and improved self-esteem, which would encourage increased social participation among older persons suffering from chronic diseases.

Nigeria’s healthcare service providers should normalize the inclusion of support groups in care plans. Health professionals should encourage the mobilization of support groups among older persons suffering from chronic illness. The existence of support groups will provide platforms for older persons to meet to discuss their struggles with one another. It is hoped that these meetings will reduce feelings of “aloneness” and despair associated with coping with chronic illness. In addition, support groups allow for sharing experiences on how people manage their conditions. Such information sharing would improve management skills and confidence in dealing with chronic illness.

### Study Limitations

Although this is the only known study exploring the coping strategies adopted by older Nigerians in dealing with chronic illness, it is important to highlight some limitations. The purposive and convenient sampling technique adopted for the study meant that only respondents who had gone to the hospital on the same days that fieldwork was conducted and who agreed to respondents were included. Further, the study had more representation of persons with low socioeconomic status than high socioeconomic status. It should be stated, however, that there was no prior knowledge of clinic days or whether any socio-economic groups were receiving treatment on those days.

Although this study’s qualitative approach might limit its generalizability, its exploratory nature provides interesting indications for further research. Given that the issue of emotional and psychological well-being resonated with our respondents, it might be interesting for future research to focus on coping strategies adopted by older Nigerians with specific chronic illnesses and the relationship between sociodemographic characteristics and depressive symptoms amongst chronically ill older adults. Furthermore, inquiries might also be focused on the perspectives of care providers and close family members of older persons suffering from chronic diseases, as well as views of community members or the general populace about the different chronic diseases. Additionally, future studies may take a more systematic approach, taking cognizance of the sociodemographic characteristics, living situation, and community characteristics of potential participants when selecting study respondents. In addition, it would be interesting to conduct research in a different setting, among older persons who use alternative healthcare services, such as with religious clerics or nonorthodox medicine.

## Conclusion

In this study, we explored the coping experiences of chronically ill Nigerian adults. We found that types of social networks, concerns about one’s physical appearance, need for privacy, and self-sufficiency affected the coping mechanism of chronically ill older adults in Nigeria. Thus, these adults rely on tools such as religion to understand and accept their chronic illness diagnoses and the financial constraints experienced due to managing their chronic illness. Also, the family was identified as the most important unit for lending instrumental, emotional, and financial support to chronically ill older adults, especially widowed older adults. The current study further illuminated how the fear of being discriminated against by others in their communities leads to the concealment of one’s illness, thereby limiting respondents’ ability to cope effectively with their diagnoses. The study draws attention to the growing need for holistic care for chronically ill older adults. Given the rising burden of chronic illnesses, as people age, it is important to enact and implement actions to alleviate chronic illness at the individual, government, and institutional levels.

## Supplementary Material

igad141_suppl_Supplementary_Material
