# Supplementary material for: The Need to Appear Healthy: Concealment of Chronic Illness, Privacy, and Self-Sufficiency Among Chronically Ill Older Nigerians
Source: Innov Aging. 2023 Dec 29;8(4):igad141. doi: 10.1093/geroni/igad141 (PMC11020260; doi:10.1093/geroni/igad141)
Supplement: igad141_suppl_Supplementary_Material [file igad141_suppl_supplementary_material.docx]

**Online Supplementary Material**

**Interview Guide**

The interview guide consists of a crib sheet to be used in the interviews, with explanatory notes to clarify the purpose of each section.

| **Interview Guide** | **Explanatory Notes** |
| --- | --- |
| Introduction  Participant Information (Scripted)  Consent (written/scripted) | See Participant Information Sheet and Consent Form |
| **Nature of Illness** | Find out about nature/type of illness of participant, whether he/she is suffering from multiple conditions, how it makes him/her feel, in what ways it is frustrating. |
| **Socio-economic status** | Find out whether participant has any form of education, level of education attainment, occupation, and ability to pay for health services received. |
| **Social support** | Whose decision it was to come to the hospital for treatment, who cares for them during this period, are any significant others (spouses, children, and other family) physically and emotionally present to render support. Do they support financially, how has this eased or increased their burden, how does this make them feel? |
| **Religion** | Find out the religious affiliation of participant, how religion has helped him/her cope; is it by increased spirituality or from social groups formed. What role(s) have the religious groups played in well-being (companionship, financial support, prayers) |
| **Community participation** | Find out if participants have friends and the role of friends in improved sense of well-being. |
| **Checklist**  **-**Age  -Tribe  -Highest educational qualification  -Occupation  -Marital status | Key factual information regarding the participant will be gathered if it has not been covered in the main body of the interview. |
